# Accounting for grouped predictor variables or pathways in high-dimensional penalized Cox regression models

**DOI:** 10.1186/s12859-020-03618-y

**Published:** 2020-07-02

**Authors:** Shaima Belhechmi, Riccardo De Bin, Federico Rotolo, Stefan Michiels

**Affiliations:** 1 Université Paris-Saclay, Univ. Paris-Sud, UVSQ, CESP, INSERM U1018 Oncostat, Villejuif, F-94805 France; 2 Service de biostatistique et d’épidémiologie, Gustave Roussy, Villejuif, F-94805 France; 3 Department of Mathematics, University of Oslo, Oslo, Norway; 4 Biostatistics and Data Management Unit, Innate Pharma, Marseille, France

**Keywords:** Lasso penalty, High-dimensional, Biomarker selection, Pathways, Cox model, Precision medicine, Stratified medicine

## Abstract

**Background:**

The standard lasso penalty and its extensions are commonly used to develop a regularized regression model while selecting candidate predictor variables on a time-to-event outcome in high-dimensional data. However, these selection methods focus on a homogeneous set of variables and do not take into account the case of predictors belonging to functional groups; typically, genomic data can be grouped according to biological pathways or to different types of collected data. Another challenge is that the standard lasso penalisation is known to have a high false discovery rate.

**Results:**

We evaluated different penalizations in a Cox model to select grouped variables in order to further penalize variables that, in addition to having a low effect, belong to a group with a low overall effect; and to favor the selection of variables that, in addition to having a large effect, belong to a group with a large overall effect. We considered the case of prespecified and disjoint groups and proposed diverse weights for the adaptive lasso method. In particular we proposed the product Max Single Wald by Single Wald weighting (MSW*SW) which takes into account the information of the group to which it belongs and of this biomarker. Through simulations, we compared the selection and prediction ability of our approach with the standard lasso, the composite Minimax Concave Penalty (cMCP), the group exponential lasso (gel), the Integrative *L*1-Penalized Regression with Penalty Factors (IPF-Lasso), and the Sparse Group Lasso (SGL) methods. In addition, we illustrated the methods using gene expression data of 614 breast cancer patients.

**Conclusions:**

The adaptive lasso with the MSW*SW weighting method incorporates both the information in the grouping structure and the individual variable. It outperformed the competitors by reducing the false discovery rate without severely increasing the false negative rate.

## Background

The development of high throughput genomic technologies has allowed for the rapid growth and easier availability of very large genomic data. These data are collected rapidly and at low cost from patients, they are usually characterized by a large number of variables or biomarkers (large *p*) and a small sample size (small *n*). Traditional variable selection methods are not well-suited for this situation: mostly based on asymptotic theory, they work well for large *n*-to-*p* ratio.

In oncology, the clinical endpoint is often a survival-type criterion and the Cox proportional hazard model [1] is typically used to estimate the effect of candidate predictor variables. In order to deal with a large number of variables an *L*_1_ penalty is often added to the model partial log-likelihood, leading to the penalized regression method lasso (least absolute shrinkage and selection operator [2, 3]). Despite the popularity of this method, it is known to select a very large number of false positives (FP) [4, 5]. Particular modifications can be used [6] to tackle this issue, for example the adaptive lasso [7, 8] that uses adaptive weights to penalize more those coefficients for which preliminary effects appear small in order to amplify the differences between the coefficient estimates.

The standard lasso method typically focuses on homogeneous data in terms of their nature and acquisition technique, and without any putative knowledge of their biological role. However, in real applications, it is increasingly common to deal with multiple sources of data, such as sequencing data, copy number variations, mutations or methylation data (data integration) or with genetic data for which the belonging to different pathways is an important information to take into account. For example, suppose we have two biomarkers groups, the first group is an active group i.e. it contains some biomarkers related to the endpoint and the second group is an inactive group i.e. it does not contain any biomarker related to the endpoint. Considering this information, it makes sense to favour the selection of the biomarkers belonging to the active group. Recently, new penalized methods have also been proposed to perform sparse group selection: for instance, the composite Minimax Concave Penalty (cMPC) [9] and the group exponential lasso (gel) [10] allow the bi-level selection of both the relevant groups and of biomarkers within the selected groups; the sparse-group lasso (SGL) [11] also allows the selection of the groups like the group lasso [12] in addition to selecting biomarkers within groups; and the Integrative *L*1-Penalized Regression with Penalty Factors method (IPF-Lasso) [13] which allows defining different penalty terms for groups of variables.

The objective of the current work was to develop a weighting strategy to integrate grouped information within the adaptive lasso framework in order to combine sparsity of groups and within groups in lasso model selection with a time-to-event outcome. The adaptive lasso method was used because of its oracle properties [14], i. e. its consistency in variable selection maintaining asymptotic efficiency in parameter estimation. Our objective was motivated by a gene expression data set of 614 patients with breast cancer treated with anthracycline-based adjuvant chemotherapy in which we wanted to perform variable selection on 128 biomarkers belonging to four important biological pathways. We propose and compare different weighting strategies for the adaptive lasso method to penalize the most those biomarkers that, in addition to having a low effect, belong to a pathway with a low overall effect; and to favor the selection of those biomarkers that, in addition to having a large effect, belong to a pathway with a large overall effect. The different propositions of weights are presented in the “Methods” section. We studied the selection and prediction ability of these models and compared them to other frequentist approaches such as the IPF-Lasso, SGL, cMCP, and gel methods through a simulations study. The proposed methods were also applied to the real cancer data set mentioned above. Our results show that the adaptive lasso with the proposed product Max Single Wald by Single Wald weighting method outperformed the other methods in terms of reducing the false discovery rate without severely increasing the false negative rate.

## Methods

In this work we focus on the selection of predictor variables or biomarkers belonging to disjoint prespecified groups. In a high-dimensional Cox model where the number of biomarkers *p* far exceeds the number of patients *n*, the lasso penalized regression is often used to select the active prognostic biomarkers (i. e. those which provide information on the overall outcome in patients) [15]. It consists in introducing an *L*_1_ penalty term in the partial log-likelihood function to allow estimation of the coefficients in the case of *p*>*n*, and to shrink toward the origin the regression coefficient estimates. The lasso penalized regression consists in maximizing the penalized partial log-likelihood function

1 $$\begin{array}{@{}rcl@{}} \ell_{p}(\beta,X) = \ell(\beta,X) \, - \, p(\lambda,\beta), \end{array} $$

with *ℓ*(*β*,*X*) the partial log-likelihood and *p*(*λ*,*β*) the *L*_1_ penalty $ \sum _{j= 1}^{p} \left |\beta _{j}\right |$. The *L*_1_ penalty shrinks each coefficient *β*_*j*_ towards zero and also sets some of them exactly to 0, which corresponds to not selecting the correspondent biomarkers.

As usually performed in practice, in this paper we choose the shrinkage parameter *λ* by maximizing the cross-validated log-likelihood function cvl [16]. We call this approach standard lasso throughout the paper, and we include it as a baseline reference in our comparisons.

### The proposed adaptive lasso

The adaptive lasso [7, 8] is an extension of the standard lasso penalty which assigns adaptive weights *W* to penalize different coefficients in the *L*_1_ penalty in such a way that small effect biomarkers are penalized more than the large effect biomarkers, in order to avoid an inaccurate selection of the noise biomarkers. The adaptive lasso estimate is given by maximisation of

2 $$\begin{array}{@{}rcl@{}} \ell_{p}(\beta,X) = \ell(\beta,X) \, - \, \lambda \sum\limits_{j= 1}^{p} \left | \beta_{j} \right | W_{j} = \ell(\beta,X) \, - \, \lambda \beta^{T} W, \end{array} $$

where $W = \left (W_{1}, \dots, W_{p}\right)^{T}$ is the biomarker-specific adaptive weights vector that can be calculated by the following propositions. Let *R* be the number of biomarker groups and *p*_*r*_ the number of biomarkers in group *r*, $r=1, \dots, R$, with $p=\sum _{r} p_{r}$. As in (1), *λ* is a shrinkage parameter that controls the amount of penalty and in this paper is computed via cross-validation.

#### Average coefficients (AC)

In a first step, for each biomarker a univariable Cox model was fitted to estimate univariable regression coefficients $\widehat \beta $. The AC consists of summarizing the information from each biomarker group using the average of the absolute values of the univariable Cox coefficients. For each biomarker *k* belonging to a given group *r*, its adaptive weight was defined as

3 $$\begin{array}{@{}rcl@{}} W_{k}^{(r)} = 1 / \left (\sum_{k= 1}^{p_{r}} \left| \widehat \beta_{k}^{(r)}\right| / p_{r}\right). \end{array} $$

Note that we use the absolute value of $\widehat \beta $ to account for both the positive and negative effects of biomarkers.

#### Principal component analysis (PCA)

PCA is also a common approach for summarizing the relevant information from a group of data. The first principal component consists of the linear combination of a group of variables with the highest variance. For each biomarker group *r*, we computed the *PC*1^(*r*)^ and we used $\widehat {\beta }^{(r)}_{PC1}$, its regression coefficient estimated in a univariable Cox model, to compute the adaptive weights

4 $$\begin{array}{@{}rcl@{}} {W_{k}}^{(r)} = 1/ \left| {\widehat \beta_{PC1}}^{(r)} \right|. \end{array} $$

#### Lasso+PCA

In order to reduce the dimension and to select the most relevant biomarkers, two steps were considered in this approach: in the first step the standard lasso was applied to all biomarkers, regardless of the group structure, in order to drop some of them out; then, the first principal component was calculated for each of group, considering only those biomarkers that were selected during the first step. If a group contained only one selected biomarker, its first principal component was the selected biomarker.

Let *R*^′^ be the number of selected (i. e. containing at least one selected biomarker) groups 1≤*R*^′^≤*R*. The univariable Cox model with the post-lasso $PC1^{(r')}, r'=1, \dots, R'$, was used to obtain the regression coefficient estimate $\widehat \beta _{Lasso+PC1}^{\left (r'\right)}$. For biomarkers in the selected groups, the adaptive weight was

5 $$\begin{array}{@{}rcl@{}} W_{k}^{\left(r'\right)} = 1 / \left|\widehat \beta_{Lasso+PC1}^{\left(r'\right)} \right|. \end{array} $$

For biomarkers in non-selected groups, the weight was equal to the maximum weight of the selected groups in order to penalize them strongly:

6 $$\begin{array}{@{}rcl@{}} W_{k}^{\left(R-R'\right)} = \underset{{~}_{1 \leq r'\leq R'}}{\max} \left(1 / \left|\widehat \beta_{Lasso+PC1}^{\left(r'\right)} \right|\right). \end{array} $$

#### Single wald (SW)

For benchmarking purposes, we included the SW weighting in this study. The SW weighting strategy assigned to each biomarker a weight equal to the inverse of its single Wald statistic from the univariable Cox model. Although this weighting does not take into account the group effect, we found it is useful to assess the additional effect of combining the SW weighting with the Average Single Wald (ASW) and Max Single Wald (MSW) (see below). The adaptive weight was then defined as

7 $$\begin{array}{@{}rcl@{}} W_{k} = 1 / SW_{k}. \end{array} $$

#### Average single wald (ASW)

We proposed a different weighting approaches based on the Wald test statistic. In the first step, for each biomarker, a univariable Cox model was fitted to estimate the single Wald statistic for testing the significance of the univariable Cox regression coefficients. To summarize information about biomarker groups, the ASW weight was defined as the average of the biomarker-associated single Wald statistics $ASW^{(r)} = \sum \limits _{k= 1}^{p_{r}} {SW}_{k}^{(r)} / p_{r}$. For each biomarker *k* belonging to the *r*^th^ group, the adaptive weight was defined as

8 $$\begin{array}{@{}rcl@{}} W_{k}^{(r)} = 1 / ASW^{(r)}. \end{array} $$

#### Max single wald (MSW)

Instead of the average of the ASW approach, we computed the maximum of the single Wald statistic [17] $MSW^{(r)} = \underset {{1 \leq k \leq p_{r}}}{\max } SW_{k}^{(r)} $. The adaptive weight was then defined as

9 $$\begin{array}{@{}rcl@{}} W_{k}^{(r)} = 1 / MSW^{(r)}. \end{array} $$

As a result, only a small penalty was applied to active groups showing some evidence of a prognostic role of any biomarker in the group, while a large penalty was applied for inactive groups.

Of note, this first weighting approach assigns different weights to the different groups, but the same weight to biomarkers within the same group. In the following, we present other weighting approaches based on Wald test statistics that assign not only different weights to biomarker groups but also to biomarkers within the same group. The rationale behind the following approaches is to combine both group-level and biomarker-level information.

#### Product of average single wald by single wald (ASW*SW)

In the ASW*SW weighting strategy, we defined the weight for each biomarker belonging to a given group *r* as the product of *ASW*^(*r*)^, the average single Wald statistic of the group, and $SW_{k}^{(r)}$, the biomarker-associated single Wald statistic. Then, the adaptive weight for biomarker *k* belonging to the *r*^th^ group was

10 $$\begin{array}{@{}rcl@{}} W_{k}^{(r)} = 1 / \left(ASW^{(r)}*SW_{k}^{(r)}\right). \end{array} $$

#### Product of max single wald by single wald (MSW*SW)

Similarly to the above strategy, the MSW*SW weighting was defined as the product of *MSW*^(*r*)^, the maximum single Wald of the group, and $SW_{k}^{(r)}$, the biomarker-associated single Wald statistic. The adaptive weight was then defined as

11 $$\begin{array}{@{}rcl@{}} W_{k}^{(r)} = 1 / \left(MSW^{(r)}*SW_{k}^{(r)}\right). \end{array} $$

These last two weightings were aimed at better discriminating between active and inactive biomarkers within active groups.

### Composite minimax concave penalty (cMCP)

The cMCP approach proposed by Breheny and Huang [9] performs selection both at the group level and at biomarker level within groups. Concurrently, it avoids overshrinkage by allowing covariates to grow large, and allows groups to remain sparse internally. The cMCP objective function takes the form

12 $$\begin{array}{@{}rcl@{}} \ell_{p}(\beta,X) = \ell(\beta,X) - \sum\limits_{r=1}^{R} f_{O}\left\{\sum_{k=1}^{p_{r}} f_{I}\left(\beta_{k}^{(r)}\right)\right\}, \end{array} $$

where *f*_*O*_=*f*_*λ*,*a*_ and *f*_*I*_=*f*_*λ*,*b*_ are the outer and the inner MCP [18], respectively. Parameters *a* and *b* are setting parameters for the inner and the outer penalties, respectively, that affect the range over which the penalty is applied.

The MCP is defined on [0,*∞*) by $f_{\lambda, a}(\beta)=\left \{\begin {array}{ll}\lambda \beta -\frac {\beta ^{2}}{2 a} & \text { if}\ \beta \leq a \lambda \\ \frac {1}{2} a \lambda ^{2} & \text { if}\ \beta >a \lambda \end {array}\right.$ for *λ*≥0. It starts by applying the same penalty rate as the lasso, and then it continuously releases this penalty until, when *β*>*aλ*, the penalty rate drops to 0.

### Group exponential lasso (gel)

The group exponential lasso [10] performs selection by maximisation of an objective function which is similar to the cMCP one, with inner penalty equal the lasso penalty, and outer penalty equal to the exponential penalty. The exponential penalty, with support [0,*∞*), is defined as *f*(*β*|*λ*,*τ*)=*λ*^2^/*τ*{1− exp(−*τβ*/*λ*)}, where *τ* is the rate of exponential decay. Of note, as *τ*→0, the gel method becomes equivalent to the standard lasso.

Although the different outer functions makes the cMCP and gel penalties different, they share similar characteristics as they are both close to the standard lasso for coefficient values close to zero, whereas they are weaker than the lasso for coefficient values far from zero.

### Sparse group lasso (SGL)

The SGL proposed by Simon et al. [11] is another method which promotes sparsity at two different levels: “groupwise sparsity” and “within group sparsity” by selecting relevant groups (in the same spirit as the lasso group [12]) and, within the selected groups, the relevant biomarkers. The objective function for the SGL method is defined as

13 $$\begin{array}{@{}rcl@{}} \ell_{p}(\beta,X) = \ell(\beta, X) \, - (1-\alpha)\lambda\sum\limits_{r= 1}^{R}\sqrt{p_{l}}||\beta^{(r)}||_{2} - \alpha\lambda||\beta||_{1}. \end{array} $$

Note that, if *α*=1 or *α*=0, the SGL penalty boils down to the standard lasso and Group Lasso, respectively. The Group Lasso method was not included in our study, because this method either selects all or none of the biomarkers in each group, which does not meet our objective of sparse model selection.

### Integrative lasso with penalty factors (IPF-Lasso)

The Integrative *L*1-Penalized Regression with Penalty Factors (IPF-Lasso) was proposed by Boulesteix et al. [13] to modify the standard lasso in the context of groups of biomarkers. The IPF-Lasso introduces different penalty factors to the different groups of variables in order to weight the data differently according to their sources. The objective function of this method is

14 $$\begin{array}{@{}rcl@{}} \ell_{p}(\beta, X)=\ell(\beta, X)-\sum\limits_{r=1}^{R} \lambda_{r}\left\|\beta^{(r)}\right\|_{1}, \end{array} $$

where the penalty factors $\lambda _{1}, \lambda _{2}, \dots, \lambda _{R}$ are chosen by cross-validation (5-fold CV and 10 repeats) via a grid search over a list of prespecified candidate vectors. In our simulations, we considered two lists of candidate penalty factors to address the effect of different specifications of this list (see below).

## Simulation study

We performed a simulation study to compare the methods outlined so far in terms of selection of prognostic biomarkers in high-dimensional Cox models and in terms of prediction accuracy. We considered the standard lasso as reference method. We used the following R packages to implement the methods described in the previous Section: glmnet [19, 20] for standard lasso and adaptive lasso; corpcor [21] for principal component analysis; grpreg [22] for cMPC and gel methods with *τ*=1/20 as suggested by the authors [10] to perform a selection of groups and within groups. We used ipflasso [23] for IPF-Lasso; SGL [24] for SGL method with the default parameter value *α*=0.95; and timeROC [25, 26] for the estimation of the area under the time-dependent ROC curve (AUC). All these packages are publicly available on the Comprehensive R Archive Network (CRAN) repository. Our code is available upon request and will be integrated in the future in the biospear package [27].

### Simulation of data

We conducted two blocks of simulations, both with *n*=500 patients and *p*=1000 biomarkers. In the first simulations (a), the *p*=1000 biomarkers were evenly distributed over *R*=20 groups of equal size *p*_*k*_=50; in the second block of simulations (b) the *R*=20 groups were of different sizes: 10 groups of *p*_*k*_=25 biomarkers and 10 groups of *p*_*k*_=75. The biomarker values were drawn from a centered and scaled (*σ*^2^=1) Gaussian distribution, with a 5-block autoregressive correlation (*ρ*=0.8^|*i*−*j*|^).

To mimic the breast cancer gene expression study of our application, an exponential survival time was generated with a baseline median survival time of eight years. The independent censoring rate was between 55% and 78%, generated from a uniform distribution U(2,6), reflecting a trial with two-year follow-up and four-year accrual.

We simulated 8 scenarios with different number values of: *q*, the number of active biomarkers (i. e. prognostic biomarkers); *l*, the number of active groups (i. e. groups containing any active biomarkers); and *HR*=*exp*(*β*), the hazard ratio for active biomarkers. For each scenario, simulations were replicated across 500 data sets. For each simulation, we generated a total of 1000 patients and we split them into a training set (*n*=500) for biomarker selection and a validation set (*n*=500) for independent estimation of the AUC.

Finally, we also consider an example in which *n*=50, to evaluate whether the results of our comparison are similar in a more extreme case.

#### Simulation scenarios

Scenarios 1-2 are the null scenarios i. e. without effects of biomarker groups. In scenario 1, the complete null scenario, there were no active biomarkers at all (*l*=0 and *q*=0); in scenario 2, all biomarker groups were active, each group containing the same number of active biomarkers (*l*=20 and *q*=100). The *HR* was randomly generated between 0.85 and 0.95 (*β*∼*U*(−0.05,−0.16)).

Scenarios 3-8 are the alternative scenarios that means that active biomarkers belonged to some particular groups. The number of active biomarkers and groups was: (*l* = 1; *q* = 8), (*l* = 2; *q* = 8), (*l* = 1; *q* = 32), (*l* = 2; *q* = 32), (*l* = 2; *q* = 16), and (*l* = 2; *q* = 8), respectively. Although, scenarios 4 and 8 have the same number of active biomarkers and groups, (*l* = 2; *q* = 8), they have a different *HR* of active biomarkers. The *HR* was randomly generated between 0.65 and 0.75 (*β*∼*U*(−0.43,−0.29)) for large effects in scenarios 3-4 and between 0.85 and 0.95 (*β*∼*U*(−0.05,−0.16)) for small effects in scenarios 5-8.

The active biomarkers were equally distributed across all active groups. The active group and biomarker index, as well as more detailed information of the simulation set-up, are shown in Additional file 1, Table 1.

#### Biomarker selection

Prespecified, uncorrelated and disjoint biomarker groups were considered, and the selection methods were applied to each generated data set. The shrinkage parameters *λ* was selected by maximising the cross-validated log-likelihood (cvl) via 5-fold cross-validation, with 10 repeats for the IPF-Lasso.

In the IPF-Lasso method, we fixed two penalty factor lists for the grid search. The first list (pflist1) assigned to one, two or three groups a penalty that was two-fold lower than all the other groups, leaving to the cross-validation process the choice of groups were the most suited to be penalized the least. The second list (pflist2) assigned to one, two or three groups a penalty that was two-fold higher than the rest of the groups, again with automatic choice of which the most suited group for the highest penalty. pflist1 = list(c(1,rep(2,19)), c(2,1,rep(2,18)), c(rep(2,10),1,rep(2,9)), c(1,1,rep(2,18)), c(1,rep(2,9),1,rep(2,9)), c(2,1,rep(2,8),1,rep(2,9)), c(1,1,rep(2,8),1,rep(2,9))). pflist2 = list(c(2,rep(1,19)), c(1,2,rep(1,18)), c(rep(1,10),2,rep(1,9)), c(2,2,rep(1,18)), c(2,rep(1,9),2,rep(1,9)), c(1,2,rep(1,8),2,rep(1,9)), c(2,2,rep(1,8),2,rep(1,9))). Since we only simulate active markers in up to two groups (the 1st and either the 2nd or the 11th, see Table 1 in the Additional file 1), these configurations allow us to investigate cases in which not enough, the correct number or too many groups get different penalties from the other. In the remaining of this paper, let IPF-Lasso1 be the IPF-lasso using the pflist1 and IPF-Lasso2 be the IPF-lasso using the pflist2.

### Evaluation criteria

The primary goal of the simulation study was to evaluate and compare the selection ability of the different methods. The primary evaluation criterion was the False Discovery Rate FDR = FP/(TP+FP) [28] of biomarkers, i. e. the rate of inactive selected biomarkers (false positives FPs) among those selected (FPs and true positives TPs), and the secondary criterion was the False Negatives Rate FNR = FN/(TP+FN) [29] of biomarkers, the rate of biomarkers excluded by the model (false negatives FNs) among the active ones (TPs and FNs). Minimizing the FDR is our primary goal, while a low FNR is a complementary measure that prevents the choice of meaningless (too sparse) models. In addition to these two measures, we calculated another exploratory criterion, the F1-score defined by 2*TP/(2*TP+FP+FN), which summarizes both the FDR and the FNR. The F1-score, also known as Dice similarity coefficient, is the geometric mean of precision and recall and ranges from 0 to 1. We also measured FDR and FNR at the group level, by extending the previous definitions as follows. We considered a group as active if and only if it contained any active biomarker and we considered a group as selected by a given method if and only if the method selected any biomarker belonging to it. The, we defined the FDR of groups as the rate of selected inactive groups among all the selected groups and the FNR of groups as the rate of non-selected groups among the active groups. In real applications, reliable selection methods are those who are characterized by a low FDR, together with as low FNR as possible (i. e. a high F1-score).

In addition to selection performances, we also assessed the predictive ability of the selection methods in terms of discrimination, i. e. the concordance between the predictor score estimated by the models and the actual survival time. To this end, we calculated the Receiving Operating Characteristic (ROC) curve and the associated Area Under Curve (AUC). As we focused on survival data, we used the Inverse Probability of Censoring Weighting (IPCW) approach, which provides consistent estimators of the ROC curve in the case of censored data [30, 31]. For each simulated data set, we computed the AUC of the selected model in the independent validation set, which was generated with the same parameters as the training data set (see above).

## Results

**Fig. 1:**
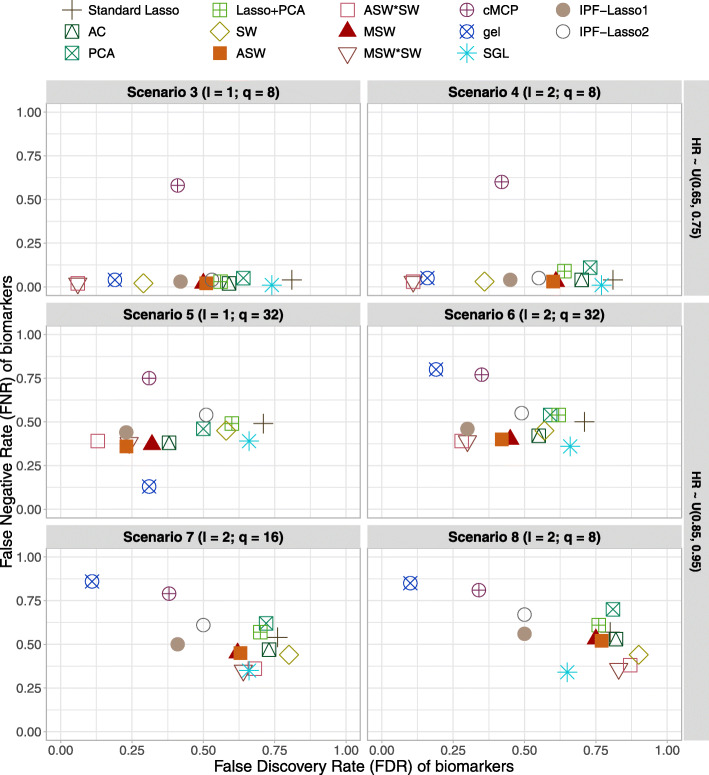
Simulation results at the biomarker level. False Discovery Rate (FDR) against False Negative Rate (FNR) of biomarkers in alternative scenarios for the simulations (a) with *n*=500 patients, *p*=1000 biomarkers and *R*=20 biomarker groups of equal size. Average quantities across 500 replications. Abbreviations: *l*, number of active groups; *q*, number of active biomarkers; AC: Average Coefficients; SW: Single Wald; ASW: Average Single Wald; ASW*SW: Average Single Wald product Single Wald; MSW: Max Single Wald; MSW*SW: Max Single Wald product Single Wald

**Fig. 2:**
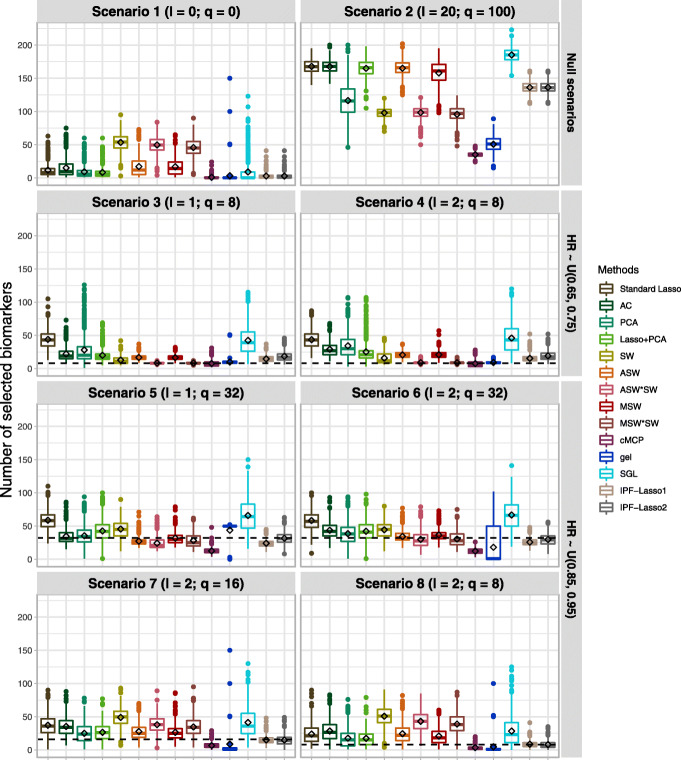
Simulation results at the biomarker level. Boxplots of the number of selected biomarkers according to the different scenarios, the box bounds the interquartile range (IQR) and contains a horizontal line corresponding to the median; outside of the box, the Tukey-style whiskers extend to a maximum of 1.5*IQR beyond the box. The black diamonds are the average number of selected biomarkers. The black dashed lines indicate the true number of active biomarkers in each scenario for the simulations (a) with *n*=500 patients, *p*=1000 biomarkers and *R*=20 biomarker groups of equal size. Abbreviations: *l*, number of active groups; *q*, number of active biomarkers; AC: Average Coefficients; SW: Single Wald; ASW: Average Single Wald; ASW*SW: Average Single Wald product Single Wald; MSW: Max Single Wald; MSW*SW: Max Single Wald product Single Wald

The results of simulations (a), for the setting of *n*=500 patients, *p*=1000 biomarkers and *R*=20 biomarker groups of equal size, are presented in this section and in Additional file 1 Table 2, Additional file 1 Table 3 and Additional file 1 Figure 1. The simulation results in Figs. 1 and 2 and in the Additional file 1 Table 2 summarize the FDR, FNR, and average number of selected biomarkers for each method over 500 replications.

In the complete null scenario 1, all selected biomarkers were false positives (i. e. there is no FNR), resulting in a mean FDR equal to the proportion of models selecting at least one biomarker. The cMCP, gel, SGL, IPF-Lasso1 and IPF-Lasso2 methods had an FDR and an average number of selected biomarkers lower than the standard lasso and adaptive lasso methods. The FDR varied from 0.25 to 0.64 for gel, cMCP, SGL and IPF-Lasso versus 1 for all other methods. The average number of selected biomarkers varied from 0.98 to for 8.99 for gel, cMCP, SGL and IPF-Lasso against higher values for all other methods except for Lasso+PCA weighting with 8.27. In the null scenario 2 with 100 prognostic biomarkers evenly distributed across the 20 groups, the gel, cMCP, SW, ASW*SW, and MSW*SW methods had a lower FDR than other methods, it was 0.05, 0.10, 0.13, 0.17, 0.18 respectively against 0.38 for IPF-Lasso1 et 0.59 for PCA weighting. The cMCP and gel methods had a lower average number of selected biomarkers than the others, 35.01 and 50.68 against values between 95.77 for MSW*SW and 185.16 for SGL.

In alternative scenarios 3 and 4 with 8 prognostic biomarkers in 1 or 2 active groups, all methods reduced the FDR compared to the standard lasso, which came with an FDR of 0.81 and an FNR of 0.04. We found that the optimal methods were ASW*SW and MSW*SW with the lowest FDR-FNR balance and average number of selected biomarkers 0.06/0.02 (8.35) for scenario 3; 0.11/0.03 (8.87) and 0.11/0.03 (8.80), respectively, for scenario 4. On the other hand, the cMCP method significantly increased the FNR compared to the standard lasso: from 0.04 to 0.58 in scenario 3 and from 0.04 to 0.60 in scenario 4. Also, cMCP selected a number of biomarkers less than the true number of active biomarkers. In Scenario 4 with two active biomarker groups (*l*=2), overall, all methods selected more biomarkers than in scenario 3 (*l*=1), and they had higher FNR. The SGL showed a marginal reduction of the FDR and increased the number of selected biomarkers to 45.88 with respect to the other methods.

In the alternative scenarios 5 and 6 with 32 prognostic biomarkers in 1 or 2 active groups, all methods reduced the FDR comparing with the standard lasso. In Scenarios 5 we noted that the ASW, ASW*SW, MSW, MSW*SW and gel methods had the best FDR-FNR balance. The number of selected biomarkers was slightly lower than the number of true active biomarkers for ASW, ASW*SW, MSW, MSW*SW, and greater than the number of true active biomarkers for the gel method. The cMCP and IPF-Lasso2 increases the FNR 0.75 and 0.54 respectively, comparing to others. In scenario 6, similarly we found that ASW, ASW*SW, MSW, MSW*SW had the best FDR-FNR balance and were also the most parsimonious ones in terms of number of selected biomarkers. Conversely, the gel method had the lowest FDR but had a number of selected biomarkers lower than the number of true active biomarkers and increased the FNR to 0.80, with a standard lasso indicating an FNR of 0.50. PCA, Lasso+PCA and SGL showing the worst results in both scenarios.

In the alternative scenarios 7 and 8 with small biomarker effects, the results were quite similar for most of the methods. The cMCP and gel methods had the lowest FDR with 0.38 and 0.11, respectively, against 0.76 for standard lasso in scenario 7 and 0.34 and 0.10, respectively, against 0.80 for standard lasso in scenario 8. But they greatly increased the FNR with 0.79 and 0.86, respectively, against 0.54 for standard lasso in scenario 7 and 0.81 and 0.85, respectively, against 0.57 for standard lasso in scenario 8. They also selected on average fewer biomarkers than the number of true active biomarkers in both scenarios. The IPF-Lasso1 method provided the best compromise between FDR and FNR (0.41 and 0.50 for scenarios 7, and 0.50 and 0.56 for scenarios 8, and the PCA weighting showing the worst results (FDR and FNR being 0.81 and 0.70).

Overall, across all scenarios, MSW and MSW*SW weighting approaches showed the best results, with the fewest selected biomarkers, the highest reduction of the FDR, and either a reduction or a non-significant increase of the FNR. In the alternative scenarios 3-6, the set of penalty factors 2 (pflist2) of the IPF-Lasso had a slightly higher FDR, FNR, and a higher number of biomarkers compared with the set of penalty factors 1 (pflist1). The adaptive lasso with PCA weighting was the only method which performed worse than the standard lasso penalty in some scenarios.

Note that, in scenarios 1, 7, and 8, the gel method had 268, 1, and 26 iterations that did not converge, respectively. All other methods had no convergence issues.

**Fig. 3:**
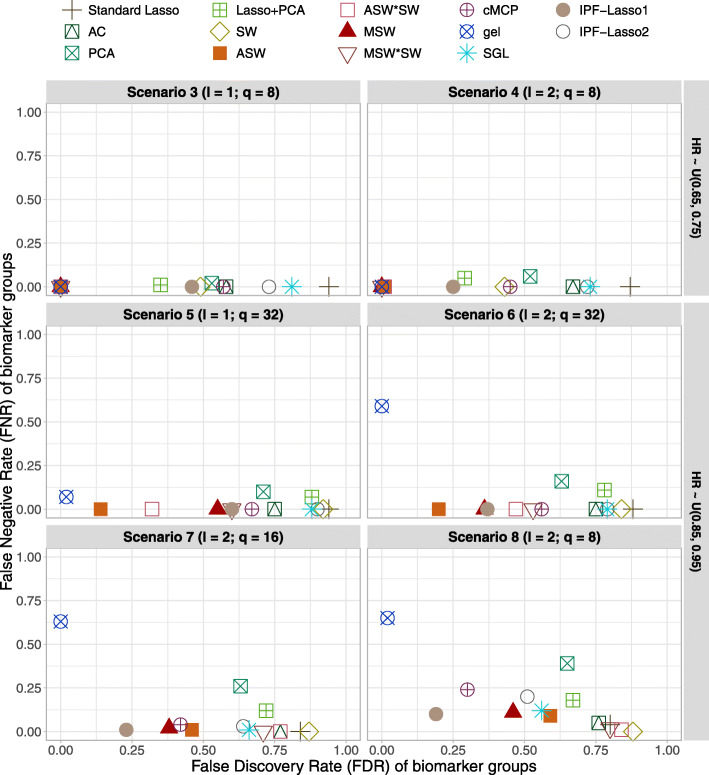
Simulation results at the group level. False Discovery Rate (FDR) against False Negative Rate (FNR) of biomarker groups in alternative scenarios for the simulations (a) with *n*=500 patients, *p*=1000 biomarkers and *R*=20 biomarker groups of equal size. Average quantities across 500 replications. Abbreviations: *l*, number of active groups; *q*, number of active biomarkers; AC: Average Coefficients; SW: Single Wald; ASW: Average Single Wald; ASW*SW: Average Single Wald product Single Wald; MSW: Max Single Wald; MSW*SW: Max Single Wald product Single Wald

Additional file 1 Table 3 and Fig. 3 summarizes the results of FDR, FNR, and number of selected biomarker groups for each method over 500 replications. In the complete null scenario 1, all selected groups were false positives (i. e. there is no FNR), resulting in a mean FDR equal to the proportion of models selecting at least one groups. The cMCP, gel, SGL, IPF-Lasso1 and IPF-Lasso2 methods had a FDR and a number of selected groups smaller than the standard lasso and adaptive lasso methods. For example, the FDR and the number of selected groups were 0.11 and 0.15 for gel and 0.64 and 1.28 for the IPF-Lasso1 and IPF-Lasso2 versus 1 and 7.55 for the standard lasso. In the null scenario 2, all groups are active (i. e. there is no FDR). Overall, the methods selected all active groups except for PCA, which had the highest FNR 0.21 and a number of selected groups 15.78 and gel method had an FNR 0.04 and number of selected groups 19.11.

In alternative scenarios 3 and 4, the ASW, ASW*SW, MSW, MSW*SW, and for gel, which had an FDR and FNR of selected biomarker groups almost equal to 0. They were also the most parsimonious in terms of the number of selected groups.

In alternative scenarios 5 and 6, we observed that all methods increased the FDR with respect to scenarios 3 and 4. Nevertheless, the ASW, ASW*SW, MSW, MSW*SW methods continued to achieve better results in terms of FDR, FNR and number of selected groups compared to the others. The gel method had the lowest FDR and the highest FNR 0.59 in scenario 6.

In alternative scenarios 7 and 8, the FDR, FNR and the number of selected groups had increased for all methods. The IPF-lasso1 had the best FDR-FNR balance. The gel method had the highest FNR of 0.65 and had a number of selected groups lower than the true number of active groups.

Table 1, shows the predictive ability of the methods in the alternative scenarios. The MSW*SW and ASW*SW methods often had the numerically highest average AUC at 5 years. The gel method had the lowest average AUC in the alternative scenarios.

**Table 1:** The average and the empirical standard error (ESE) of the AUC at 5 years in alternative scenarios for the simulations (a) with *n*=500 patients, *p*=1000 biomarkers and *R*=20 biomarker groups of equal size. Average quantities across 500 replications

**Methods**	**Scenario 3**	**Scenario 4**	**Scenario 5**	**Scenario 6**	**Scenario 7**	**Scenario 8**
	(*l* = 1; *q* = 8)	**(***l* = 2; *q* = 8)	**(*l* = 1;*q* = 32)**	**(*l* = 2;*q* = 32)**	**(*l* = 2;*q* = 16)**	**(*l* = 2;*q* = 8)**
	HR ∼*U*(0.65,0.75)		HR ∼*U*(0.85,0.95)			
Standard Lasso	0.88 (0.02)	0.87 (0.03)	0.75 (0.04)	0.75 (0.04)	0.67 (0.05)	0.61 (0.05)
Adaptive lasso						
AC	0.88 (0.02)	0.88 (0.03)	0.78 (0.04)	0.77 (0.04)	0.68 (0.05)	0.61 (0.05)
PCA	0.87 (0.06)	0.85 (0.05)	0.74 (0.09)	0.73 (0.07)	0.64 (0.07)	0.58 (0.06)
Lasso+PCA	0.88 (0.04)	0.86 (0.05)	0.74 (0.08)	0.73 (0.06)	0.66 (0.06)	0.61 (0.06)
SW	0.88 (0.02)	0.88 (0.03)	0.75 (0.04)	0.75 (0.04)	0.65 (0.05)	0.59 (0.05)
ASW	0.88 (0.02)	0.88 (0.03)	0.78 (0.03)	0.77 (0.04)	0.68 (0.05)	0.61 (0.05)
ASW*SW	0.89 (0.02)	0.88 (0.02)	0.78 (0.04)	0.77 (0.04)	0.66 (0.05)	0.59 (0.05)
MSW	0.88 (0.02)	0.88 (0.02)	0.78 (0.04)	0.77 (0.04)	0.68 (0.05)	0.62 (0.05)
MSW*SW	0.89 (0.02)	0.88 (0.02)	0.77 (0.04)	0.76 (0.04)	0.67 (0.05)	0.60 (0.05)
cMCP	0.87 (0.03)	0.86 (0.03)	0.75 (0.04)	0.74 (0.04)	0.67 (0.05)	0.61 (0.06)
gel	0.88 (0.02)	0.88 (0.02)	0.74 (0.08)	0.60 (0.08)	0.58 (0.07)	0.57 (0.06)
SGL	0.88 (0.02)	0.88 (0.02)	0.77 (0.04)	0.76 (0.04)	0.69 (0.05)	0.62 (0.06)
IPF-Lasso						
IPF-Lasso1	0.88 (0.02)	0.88 (0.03)	0.78 (0.04)	0.77 (0.04)	0.69 (0.05)	0.63 (0.05)
IPF-Lasso2	0.88 (0.02)	0.88 (0.03)	0.76 (0.04)	0.75 (0.04)	0.68 (0.05)	0.62 (0.06)

Abbreviations: *l*, number of active groups; *q*, number of active biomarkers; AC: Average Coefficients; SW: Single Wald; ASW: Average Single Wald; ASW*SW: Average Single Wald product Single Wald; MSW: Max Single Wald; MSW*SW: Max Single Wald product Single Wald.

IPF-Lasso1: pflist1 = list(c(1,rep(2,19)), c(2,1,rep(2,18)), c(rep(2,10),1,rep(2,9)), c(1,1,rep(2,18)), c(1,rep(2,9),1,rep(2,9)), c(2,1,rep(2,8),1,rep(2,9)), c(1,1,rep(2,8),1,rep(2,9))).

IPF-Lasso2: pflist2 = list(c(2,rep(1,19)), c(1,2,rep(1,18)), c(rep(1,10),2,rep(1,9)), c(2,2,rep(1,18)), c(2,rep(1,9),2,rep(1,9)), c(1,2,rep(1,8),2,rep(1,9)), c(2,2,rep(1,8),2,rep(1,9)))

The full results of simulations (b), for the setting of *n*=500 patients, *p*=1000 biomarkers and *R*=20 biomarker groups of different sizes: 10 of size 25 and 10 of size 75, are provided in the Additional file 1. The results of the simulations (b) were consistent with the results of the simulations (a). In summary, the ASW, ASW*SW, MSW, MSW*SW weighting had the best FDR-FNR balance, except for the alternative scenarios 7 and 8, where the IPF-Lasso1 showed a good results in terms of FDR-FNR balance. We observed that the gel method had a low FDR of biomarkers (or groups) but it increased the FNR of biomarkers (or groups) compared to the standard lasso and generally selected few biomarkers (or groups). Additional file 1, Table 4 also showed that all methods provided rather similar average AUC at 5 years.

Similarly to before, in scenarios 1, 7, and 8, the gel method had 327, 6, and 27 iterations that did not converge, respectively.

The F1-score results for simulations (a) and (b) are presented in heatmaps together with FDR, FNR in Additional file 1 Figure 6-17 at the biomarker level and at the group level. The results show that the adaptive lasso with double weight (at individual and group level) is uniformly in the top-3 of the best performing methods. The cMCP and gel methods have in general very low FDR, but with a sensibly increased FNR, which reflects into a F1-score which is at best in the average of all methods. Conversely, the SGL method has low FNR in general, but with relatively high FDR and then poor F1-score. In terms of selection of groups, the adaptive lasso with only group penalties perform the best, while the double penalties remain competitive.

In a small high-dimensional sensitivity study, we created a more extreme case on the same dataset of simulation (a) with the number of biomarkers *p*=1000 but only with a sample size *n*=50 for the standard lasso and adaptive lasso methods. The Table 9 of results presented in the Additional file 1 shows that, overall, the FDR and FNR increased and that the MSW*SW method has a lower FDR than the standard lasso.

## Application on real data

We applied the methods discussed so far to gene expression data of 614 patients with breast cancer treated with anthracycline-based chemotherapy. The breast cancer gene expression data set is publicly available in the biospear R package. We picked four biological pathways from a previous publication on the prognostic role of pathways in early breast cancer [32], in which pathways were modeled as weighted averages of the genes included. We choose 3 pathways with a suggested prognostic effect in that paper (Immune System, Proliferation and Stroma invasion) and 1 without (SRC activation pathway). The standardized genes in the data set analysed belong to disjoint pathways : “Immune System” (53 genes), “Proliferation” (44 genes), “Stroma” (19 genes), and “SRC” (12 genes). The purpose of this application was to identify potentially prognostic genes in pathways presumed to play a prognostic role in breast cancer, by favouring genes in the pathways showing the strongest overall evidence of containing prognostic information. The 5-year relapse-free survival rate in these patients was estimated to 74% [95% CI: 69% -77%]. For the IPF-Lasso method, we chose a penalty factor list pflist penalizing one or two groups as twice or as half as the others: pflist = list(c(1,2,2,2), c(2,1,2,2), c(2,2,1,2), c(2,2,2,1), c(2,1,1,1), c(1,2,1,1), c(1,1,2,1), c(1,1,1,2), c(1,1,2,2), c(1,2,1,2), c(1,2,2,1), c(2,1,1,2), c(2,1,2,1), c(2,2,1,1)). From the gene expression data, we performed 500 random split into training and validation sets 60% and 40% of samples, respectively. We applied the variables selection procedures on each training set and estimated the AUC for each associated validation set.

**Fig. 4:**
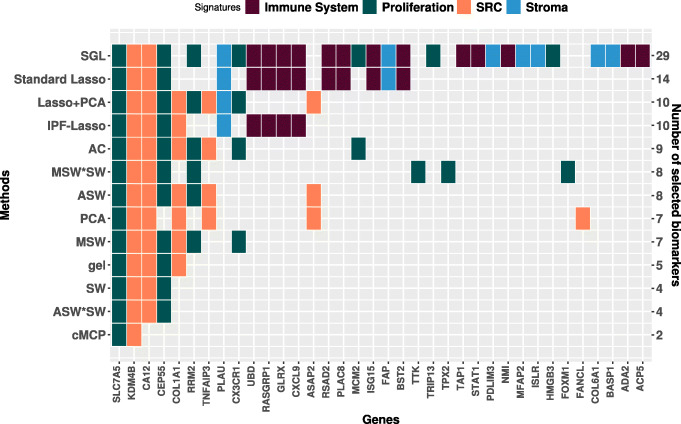
The identified biomarkers by the different lasso-based variable selection procedures on the training set of the breast cancer gene expression study for relapse-free survival. Abbreviations: AC, Average Coefficients; SW, Single Wald; ASW, Average Single Wald; ASW*SW, Average Single Wald product Single Wald; MSW, Max Single Wald; MSW*SW, Max Single Wald product Single Wald

The results of the application of the lasso-based procedures on the training data set across the 500 repetitions are summarized in Fig. 4. The figure reports the genes identified at least more than 10% of 500 repetitions and the number of biomarkers identified by each method. Figure 4 shows that the genes that belong to the Proliferation group were selected more frequently by several methods than those in the SRC, the Immune System and Stroma groups. We observed some variation between the methods in terms of the number of selected biomarkers: the SGL method provided the less sparse solution, with 29 genes selected (2 in SRC; 7 in Stroma; 7 in Proliferation; 13 in Immune System). The standard lasso reference method selected 14 genes (2 in SRC; 2 in Stroma; 2 in Proliferation; 8 in Immune System). The IPF-Lasso method selected 10 genes (1 in Stroma; 3 in SRC; 2 in Proliferation; 4 in Immune System). The cMCP selected only 2 genes belonging to the SRC and Proliferation signatures (SLC7A5, KDM4B). The gel method selected 5 genes (2 in Proliferation; 3 in SRC) (SLC7A5, KDM4B, CA12, CEP55, COL1A1).

Our proposed method, the adaptive lasso with MSW weighting selected 7 genes (3 in SRC; 4 in Proliferation) and the ASW weighting selected 8 genes (3 in Proliferation; 5 in SRC). The MSW*SW weightings selected 8 genes of which 2 genes belonging to the SRC (KDM4B, CA12) and 6 belonging to the Proliferation signature, previously suggested to be the most prognostic pathway in early breast cancer [33] (SLC7A5, CEP55, RRM2, TTK, TPX2, FOXM1), and the SW and ASW*SW weightings selected 4 genes of which 2 genes belonging to the SRC (KDM4B, CA12) and 2 belonging to the Proliferation signature (SLC7A5, CEP55).

We evaluated the AUC of 5-year relapse-free survival prediction for all methods based on the available gene expression data. Table 2 displays the average and the empirical standard error (ESE) of the AUC across 500 random split training-validation sets. Our competitor, the standard lasso, obtained an average AUC of 0.61 with an ESE of 0.04. In view of the ESE values, differences in AUC seemed small between the methods. The adaptive lasso with Lasso+PCA and PCA weighting provided the highest pointwise average AUC of 0.63 (ESE 0.04). The SGL method provided an AUC of 0.62 (ESE 0.04), and the adaptive lasso with MSW weighting an AUC of 0.61 (ESE 0.04). The gel method yielded the lowest AUC 0.57 (ESE 0.05) and the cMCP method an AUC of 0.60 (ESE 0.05). The adaptive lasso using weighting schemes AC, ASW, SW, MSW*SW, and ASW*SW provided a similar AUC 0.62 (ESE 0.04) as compared to the standard lasso technique.

**Table 2:** The average AUC and the empirical standard error (ESE) for 5-year relapse-free survival prediction obtained from 500 random training-validation sets using the different variables selection procedures

	**SGL**	**Standard lasso**	**Lasso+PCA**	**IPF-Lasso**	**AC**	MSW*SW	**ASW**	**PCA**	**MSW**	**gel**	**SW**	ASW*SW	**cMCP**
AUC	0.62	0.61	0.63	0.61	0.62	0.62	0.62	0.63	0.61	0.57	0.62	0.62	0.60
ESE	0.04	0.04	0.04	0.05	0.04	0.04	0.04	0.04	0.04	0.05	0.04	0.04	0.05

## Discussion

In the context of precision medicine and high-dimensional data, a common problem is how to take into account pathways or groups of biomarkers in the variable selection procedure. A standard method for the selection of biomarkers from a high-dimensional set of candidates is to subtract a *L*_1_ penalty from the partial log-likelihood in the Cox model [2, 3]. Nevertheless, there exist to date few methods to account for grouped information in lasso selection. In this paper, we proposed different weighting strategies for the adaptive lasso [14] to identify the active biomarkers belonging to disjoint prespecified pathways or groups for a time-to-event outcome, by favouring selection of biomarkers belonging to groups that provide the most prognostic information overall. The rationale biomarker selection can be improved by leveraging information at the group level.

In our simulations, we compared head to head our proposed approach based on the adaptive lasso to the cMCP, gel, SGL, and IPF-Lasso methods in terms of variable selection and prediction performances. Based on the results of the simulations study, the adaptive lasso with product Max Single Wald by Single Wald (MSW*SW) and adaptive lasso with product Average Single Wald by Single Wald (ASW*SW) weighting methods outperform their competitors with regards of the FDR-FNR balance both in terms of biomarkers and in terms of groups. In addition, these methods were the most parsimonious ones in terms of number of selected biomarkers. Moreover, in terms of prediction performance, the MSW*SW and ASW*SW had often the numerically highest average AUC.

Despite the cMCP and gel methods showing a low FDR, these methods had higher FNR than the standard lasso. The SGL method showed the worst results and selected a high number of biomarkers in general. Conversely, the MCP and gel methods often provided parsimonious solutions. In terms of prediction, the gel method showed poorer performances than the other methods.

Although the IPF-Lasso method showed good performances in some alternative scenarios, this approach needs to arbitrarily prespecify the list of candidate weights to be tested. This task requires quite strong knowledge or belief about the possible prognostic roles of different groups. As the number of groups increases, this limitation can become serious and the impact of such choice on the result can be strong.

In this paper, in our comparison study we fixed one on the values of the additional parameter for the SGL and gel methods. In order to verify this does not disadvantages these methods, we performed a sensitivity analysis on the same simulation data set (a) with using an additional cross-validation loop for the choice of the tuning parameters across a grid of values, i.e. for *α* in the SGL method and *τ* in the gel method. The results of simulations presented in the Additional file 1 Tables 7 and 8 show that there is not a strong difference between the results obtained by SGL with using cross-validation for the choice of *α*, and the results obtained by SGL with the fixed value of *α*, presented in the Additional file 1 Tables 2 and 3. Additional file 1 Tables 7 and 8 show that the gel method, with cross-validation for *τ* selection, gave poorer results (with a higher FDR, number of selected biomarkers and number of selected groups) than the gel method with the fixed *τ* value.

Recently, Tang et al. proposed a Bayesian method called Gsslasso Cox [34]. It is a Bayesian hierarchical model for predicting survival and detecting associated genes by incorporating pathway information. They compared their method with frequentist alternatives such as the standard lasso, SGL and cMCP. In their simulation study, they found that cMCP was consistently better than the other frequentist approaches. In contrast, in our simulation study, cMCP showed poorer performance than our proposed MSW*SW method.

In the present paper, we limited ourselves to lasso-based selection regression in Cox models. Other approaches like component-wise boosting [35] and Sparse Partial Least Squares (Sparse PLS) [36] could be considered, but are out of the scope of the present work. Similarly, while we only considered frequentist approaches, the a priori knowledge about biomarker groups could fit a Bayesian approach [37] with a hierarchical structured variable selection (HSVS) method.

Finally, we focused on uncorrelated and disjoint groups of biomarkers but the methods presented here can be extended to the case of overlapping groups (i. e. with biomarkers belonging to several groups) by biomarker duplication [38]. We considered all along the paper the example of genes belonging to functional pathways, the same methods apply to a broader range of applications, e. g. to the case of different set of genomic data from distinct nature and sources.

## Conclusion

When fitting high-dimensional penalized Cox regression models, the selection of biomarkers can take advantage of their membership to prespecified groups with a putative biological role. We have proposed an approach based on the adaptive lasso, with different weighting strategies including those based on Wald test statistics. In particular, we advocate the use of the MSW*SW weighting scheme, which, by weighting each biomarker differently, takes into account both the prognostic role of the biomarker groups and the individual prognostic role of each biomarker. In our simulations, this method outperformedss the competitors by reducing the FDR without severely increasing the FNR.

## Supplementary information

**Additional file 1** Additional documents and results of the simulation study.

## Data Availability

The breast cancer gene expression data set is publicly available in the biospear R package https://cran.r-project.org/web/packages/biospear/biospear.pdf. The program code of the proposed approach will be made available in the biospear R package.

## Abbreviations

Lasso: least absolute shrinkage and selection operator
AC: Average Coefficients
PCA: Principal Component Analysis
SW: Single Wald
ASW: Average Single Wald
ASW*SW: Average Single Wald product Single Wald
MSW: Max Single Wald
MSW*SW: Max Single Wald product Single Wald
cMCP: composite Minimax Concave Penalty
gel: group exponential lass
SGL: Sparse Group Lasso
IPF-Lasso: Integrative L1-Penalized Regression with Penalty Factors
FP: False positives
TP: true positives
FDR: False Discovery Rate
FNR: False Negatives Rate
AUC: Area under curve
