# Supplementary material for: Accounting for grouped predictor variables or pathways in high-dimensional penalized Cox regression models
Source: BMC Bioinformatics. 2020 Jul 2;21:277. doi: 10.1186/s12859-020-03618-y (PMC7331150; doi:10.1186/s12859-020-03618-y)
Supplement: Supplementary file 1 — Additional file 1 Additional documents and results of the simulation study. [file 12859_2020_3618_MOESM1_ESM.zip › boxplot_groups_v.pdf]

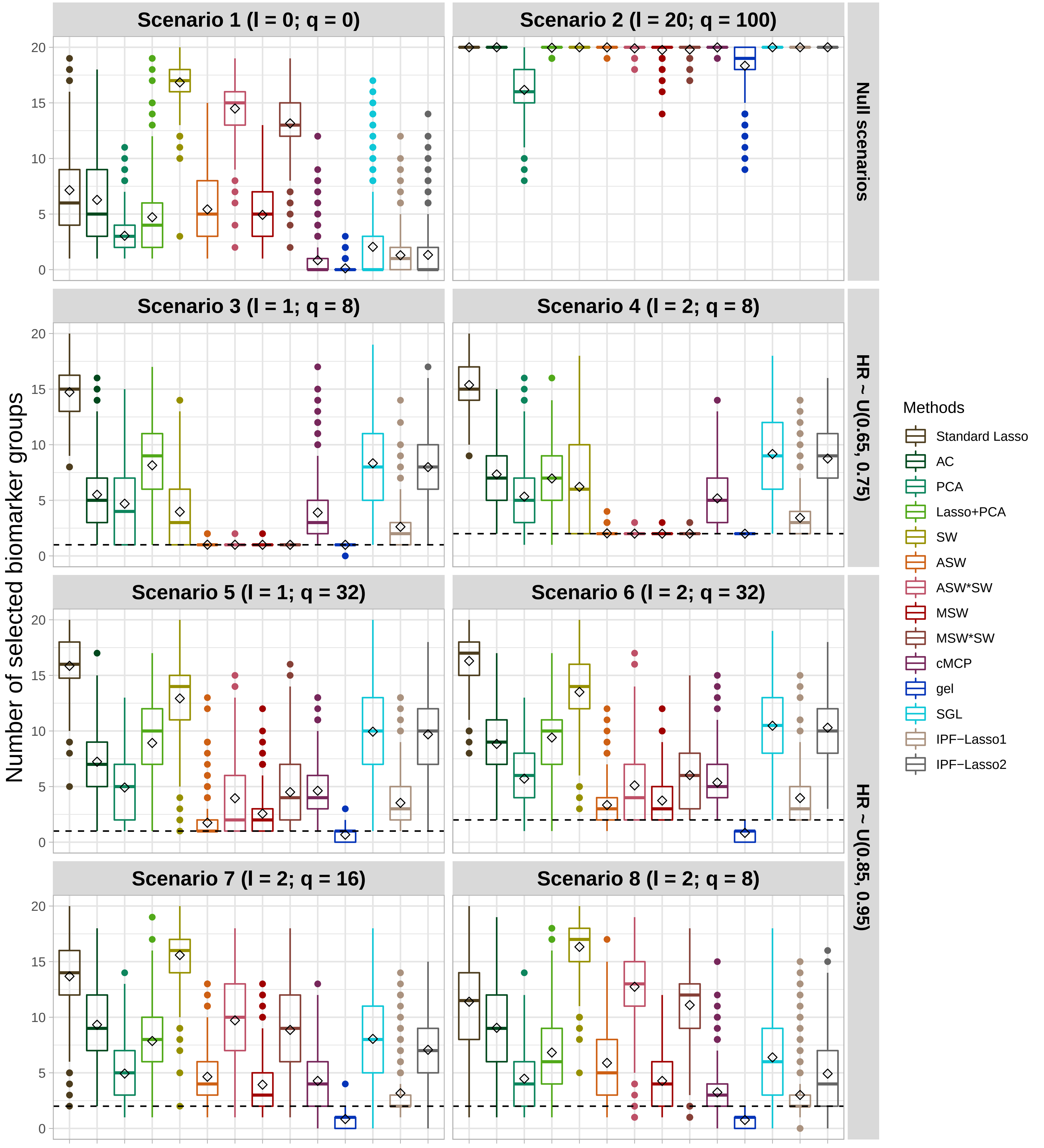
Scenario 1 (l = 0; q = 0)
Scenario 2 (l = 20; q = 100)
Null scenarios
Scenario 3 (l = 1; q = 8)
Scenario 4 (l = 2; q = 8)
HR ~ U(0.65, 0.75)
Scenario 5 (l = 1; q = 32)
Scenario 6 (l = 2; q = 32)
Scenario 7 (l = 2; q = 16)
Scenario 8 (l = 2; q = 8)
HR ~ U(0.85, 0.95)
Number of selected biomarker groups
0
5
10
15
20
Methods
Standard Lasso
AC
PCA
Lasso+PCA
SW
ASW
ASW*SW
MSW
MSW*SW
cMCP
gel
SGL
IPF−Lasso1
IPF−Lasso2
