# Supplementary material for: Accounting for grouped predictor variables or pathways in high-dimensional penalized Cox regression models
Source: BMC Bioinformatics. 2020 Jul 2;21:277. doi: 10.1186/s12859-020-03618-y (PMC7331150; doi:10.1186/s12859-020-03618-y)
Supplement: Supplementary file 1 — Additional file 1 Additional documents and results of the simulation study. [file 12859_2020_3618_MOESM1_ESM.zip › tabf1_biom_a.pdf]

	Scenario									
	2	3	4	5	6	7	8	Med	Min	Max
Standard Lasso	0.29	0.32	0.31	0.36	0.36	0.29	0.24	0.31	0.24	0.36
AC	0.64	0.57	0.45	0.61	0.50	0.34	0.23	0.50	0.23	0.64
PCA	0.43	0.51	0.39	0.51	0.41	0.29	0.20	0.41	0.20	0.51
Lasso+PCA	0.62	0.59	0.49	0.44	0.40	0.33	0.26	0.44	0.26	0.62
SW	0.85	0.80	0.73	0.46	0.47	0.29	0.17	0.47	0.17	0.85
ASW	0.62	0.65	0.56	0.69	0.58	0.42	0.27	0.58	0.27	0.69
ASW*SW	0.82	0.96	0.92	0.70	0.64	0.40	0.21	0.70	0.21	0.96
MSW	0.58	0.65	0.55	0.65	0.57	0.43	0.30	0.57	0.30	0.65
MSW*SW	0.80	0.96	0.93	0.67	0.63	0.44	0.25	0.67	0.25	0.96
cMCP	0.46	0.46	0.43	0.36	0.33	0.29	0.26	0.36	0.26	0.46
gel	0.63	0.87	0.89	0.68	0.16	0.14	0.18	0.63	0.14	0.89
SGL	0.66	0.39	0.36	0.42	0.43	0.41	0.36	0.41	0.36	0.66
IPF−Lasso1	0.71	0.71	0.68	0.64	0.60	0.53	0.43	0.64	0.43	0.71
IPF−Lasso2	0.71	0.62	0.60	0.46	0.47	0.41	0.33	0.47	0.33	0.71
